# Basis and Design of a Randomized Clinical Trial to Evaluate the Effect of Levosulpiride on Retinal Alterations in Patients With Diabetic Retinopathy and Diabetic Macular Edema

**DOI:** 10.3389/fendo.2018.00242

**Published:** 2018-05-29

**Authors:** Ma. Ludivina Robles-Osorio, Renata García-Franco, Carlos D. Núñez-Amaro, Ximena Mira-Lorenzo, Paulina Ramírez-Neria, Wendy Hernández, Ellery López-Star, Thomas Bertsch, Gonzalo Martínez de la Escalera, Jakob Triebel, Carmen Clapp

**Affiliations:** ^1^Facultad de Ciencias Naturales, Universidad Autónoma de Querétaro (UAQ), Querétaro, Mexico; ^2^Instituto Mexicano de Oftalmología, I.A.P., Querétaro, Mexico; ^3^Instituto de Neurobiología, Universidad Nacional Autónoma de México (UNAM), Querétaro, Mexico; ^4^Institute for Clinical Chemistry, Laboratory Medicine and Transfusion Medicine, Nuremberg General Hospital & Paracelsus Medical University, Nuremberg, Germany

**Keywords:** dopamine receptor type 2 blockers, prolactin, vasoinhibins, ranibizumab, retina, microvascular alterations, pituitary hormones, peptide hormones

## Abstract

**Background:**

Diabetic retinopathy (DR) and diabetic macular edema (DME) are potentially blinding, microvascular retinal diseases in people with diabetes mellitus. Preclinical studies support a protective role of the hormone prolactin (PRL) due to its ocular incorporation and conversion to vasoinhibins, a family of PRL fragments that inhibit ischemia-induced retinal angiogenesis and diabetes-derived retinal vasopermeability. Here, we describe the protocol of an ongoing clinical trial investigating a new therapy for DR and DME based on elevating the circulating levels of PRL with the prokinetic, dopamine D2 receptor blocker, levosulpiride.

**Methods:**

It is a prospective, randomized, double-blind, placebo-controlled trial enrolling male and female patients with type 2 diabetes having DME, non-proliferative DR (NPDR), proliferative DR (PDR) requiring vitrectomy, and DME plus standard intravitreal therapy with the antiangiogenic agent, ranibizumab. Patients are randomized to receive placebo (lactose pill, orally TID) or levosulpiride (75 mg/day orally TID) for 8 weeks (DME and NPDR), 1 week (the period before vitrectomy in PDR), or 12 weeks (DME plus ranibizumab). In all cases the study medication is taken on top of standard therapy for diabetes, blood pressure control, or other medical conditions. Primary endpoints in groups 1 and 2 (DME: placebo and levosulpiride), groups 3 and 4 (NPDR: placebo and levosulpiride), and groups 7 and 8 (DME plus ranibizumab: placebo and levosulpiride) are changes from baseline in visual acuity, retinal thickness assessed by optical coherence tomography, and retinal microvascular abnormalities evaluated by fundus biomicroscopy and fluorescein angiography. Changes in serum PRL levels and of PRL and vasoinhibins levels in the vitreous between groups 5 and 6 (PDR undergoing vitrectomy: placebo and levosulpiride) serve as proof of principle that PRL enters the eye to counteract disease progression. Secondary endpoints are changes during the follow-up of health and metabolic parameters (blood pressure, glycated hemoglobin, and serum levels of glucose and creatinine). A total of 120 patients are being recruited.

**Discussion:**

This trial will provide important knowledge on the potential benefits and safety of elevating circulating and intraocular PRL levels with levosulpiride in patients with DR and DME.

**Ethics and dissemination:**

Ethics approval has been obtained from the Ethics Committees of the National University of Mexico (UNAM) and the Instituto Mexicano de Oftalmología, I.A.P. Dissemination will include submission to peer-reviewed scientific journals and presentation at congresses.

**Clinical trial registration:**

Registered at www.ClinicalTrials.gov, ID: NCT03161652 on May 18, 2017.

## Background

Diabetic retinopathy (DR) and diabetic macular edema (DME) are leading causes of blindness in adults under 75 years, and nearly all type 1 diabetic patients and more than 60% of type 2 diabetes patients have evidence of DR 15–20 years after diagnosis (1, 2). DR damages the integrity of retinal microvessels and progresses from a nonproliferative (NPDR) to a proliferative vascular stage (PDR). NPDR begins with abnormal capillary permeability and exudative changes that impair vision when the macula is affected (DME). PDR develops when occlusion of retinal capillaries creates areas of ischemia that promote the proliferation of blood vessels (angiogenesis) in the surface of the retina. The new blood vessels impair vision by bleeding into the vitreous, and ultimately lead to blindness by causing traction retinal detachment. Clinically important risk factors for progression to vision loss include hyperglycemia, hypertension, and dyslipidemia (1). However, the combined values of these parameters account for only 10% of the risk of DR (3–5). Laser therapy is partially effective for preserving sight, destroys retinal tissue, and is poor for reversing visual loss (6). Anti-angiogenic therapies are effective and less destructive but require frequent intravitreal delivery, which raises the risk of infection and ocular complications (7). Therefore, the prevention and treatment of DR and DME should explore other modifiable factors and less invasive modes of administration. The current trial investigates the potential benefits and safety of elevating the serum levels of prolactin (PRL) by the oral administration of the prokinetic, dopamine D2 receptor blocker, levosulpiride.

Prolactin, the pituitary hormone essential for lactation, can protect against DR and DME *via* its proteolytic conversion to vasoinhibins, a family of PRL fragments that inhibit the proliferation, permeability, and dilation of blood vessels (8). The generation of vasoinhibins regulated at the hypothalamus, the pituitary, and the target tissue levels defines the PRL/vasoinhibin axis (9). This axis participates in maintaining corneal avascularity (10) and normal retinal vasculature (11), and is altered in retinopathy of prematurity (12) and in DR (13). Vasoinhibins are reduced in the circulation of patients with DR (14) and preclinical studies show that raising systemic PRL levels leads to vasoinhibin accumulation in the retina (15). The elevation of intraocular vasoinhibins inhibits ischemia-induced retinal angiogenesis (16) and prevents and reverses diabetes-induced blood retinal barrier breakdown by targeting excessive vasopermeability (15, 17–19) and the outer component of the blood retinal barrier (retinal pigment epithelial cells) (20). Moreover, retinal neurodegeneration influences DR (4, 21) and PRL, itself, is a retinal trophic factor. Raising circulating PRL levels reduces retinal cell death and dysfunction in the continuous light-exposure model of retinal degeneration (22). Therefore, it is hypothesized that medications causing hyperprolactinemia result in increased ocular PRL and vasoinhibins levels with beneficial outcomes in DR and DME, because of the antagonizing properties of both hormones on diabetes-induced retinal alterations.

Levosulpiride is an effective medication for inducing hyperprolactinemia (23). It works as potent prokinetic in dyspeptic syndromes including diabetic gastroparesis, a complication found in 5% of diabetic patients (24, 25). The prokinetic effect of levosulpiride is mediated through blockage of enteric inhibitory dopaminergic D2 receptors, and D2 receptor antagonism at the anterior pituitary level evokes hyperprolactinemia (26).

A randomized, double-blind, placebo-control trial has been implemented with the aim to investigate the therapeutic potential and safety of levosulpiride in patients with DR and in DME patients treated or not with standard antiangiogenic therapy (ranibizumab). This paper describes the methodology and specific details underlying the study.

## Methods/Design

### Study Setting and Sample

A total of 120 patients will be enrolled in this study over the course of 3 years. Recruitment takes place at an out-patient clinical Institution [Mexican Institute of Ophthalmology, Private Assistance Institution (Instituto Mexicano de Oftalmología, I.A.P.), Queretaro, Mexico] at the time of a routine health care or with patients referred from other out-patient clinics and health facilities. The principal investigators or investigators inform the patients (orally and in writing) about benefits and risks of participating in the study and of the clinical assessments and randomized allocation of treatment. Patients have to provide written consent before being enrolled and prior the initiation of any study-related assessment. Patients may withdraw their consent to participate at any time without providing a reason. There is no financial compensation for the study participation. However, all clinical evaluations and study medication have no cost for the patients.

Based on the current status, we expect a screening rate of 600 patients in 3 years, out of which about 120 are expected to meet the inclusion criteria, be willing to participate and, therefore, enrolled in the study. The dropout rate is expected to be less than 20%. The study may expand to a multicenter format if not enough patients are recruited.

### Study Design

**Figure 1:**
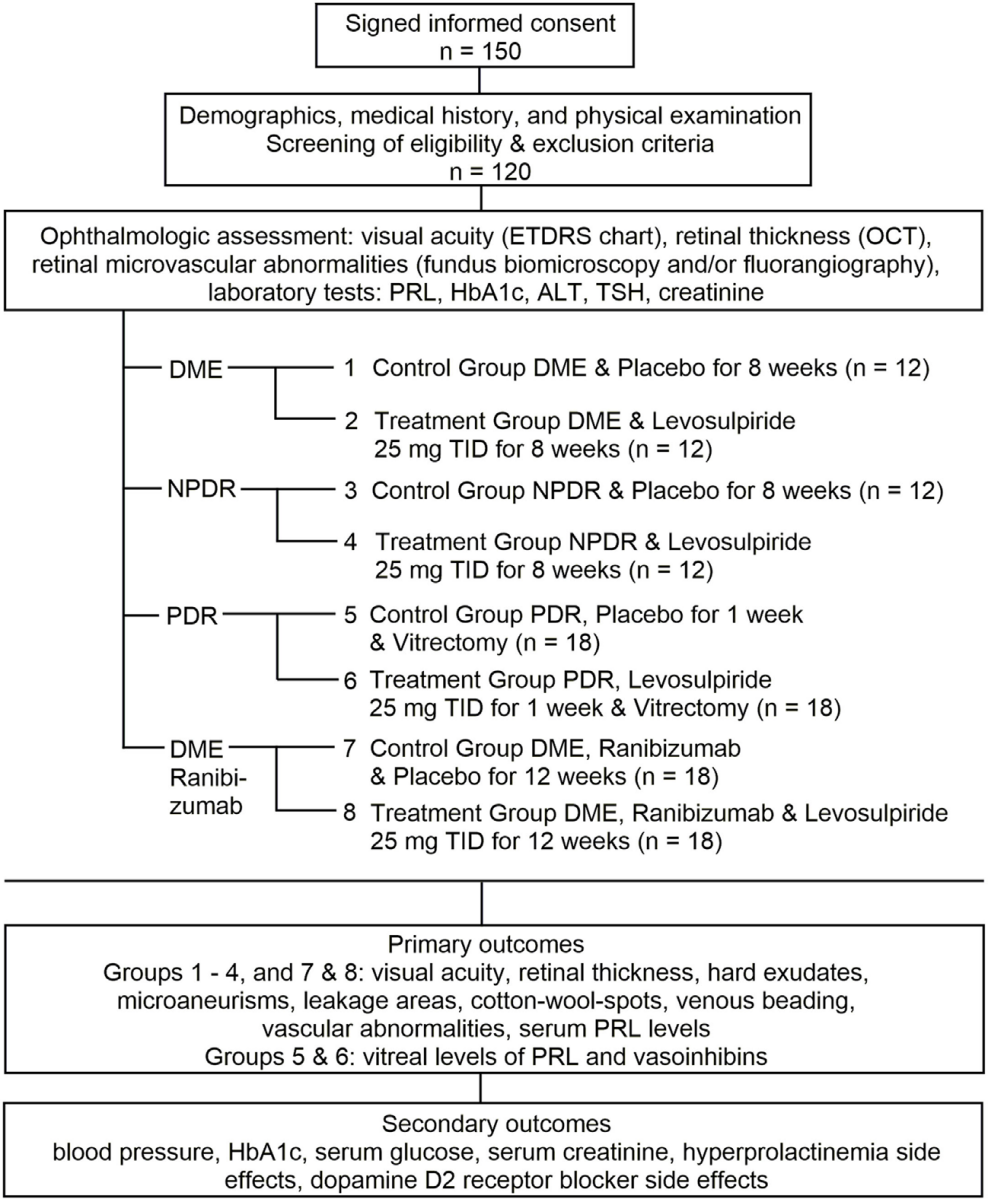
Study design. ETDRS, Early Treatment Diabetic Retinopathy Study; OCT, optical coherence tomography; HbA1c, glycated hemoglobin; ALT, alanine aminotransferase; TSH, thyroid-stimulating hormone; DME, diabetic macular edema; TID, three times a day; NPDR, non-proliferative diabetic retinopathy; PDR, proliferative diabetic retinopathy.

The study design is shown in Figure 1. This is a single-center, prospective, randomized, placebo-controlled, double-blind clinical trial on the efficacy and safety of levosulpiride for the treatment of DR and DME. An eight-armed study design is followed in which diabetic male and female subjects are selected from four different populations, those having DME, NPDR, PDR undergoing vitrectomy, and DME that will receive antiangiogenic therapy with ranibizumab. Ranibizumab is a humanized monoclonal antibody fragment directed against all forms of vascular endothelial growth factor (VEGF) approved as antiangiogenic therapy for DME (27). Immediately after baseline, each of the four groups is randomly split up into a subgroup that receives levosulpiride and a subgroup that receives placebo (lactose pill). Allocation is randomly assigned *via* computer-generated random numbers. The comparison of ophthalmologic and health outcomes between groups 1 and 2 (DME: placebo and levosulpiride) and groups 3 and 4 (NPDR: placebo and levosulpiride) will evaluate the efficacy and safety of the medication against DME and NPDR, respectively. Comparison between groups 7 and 8 (DME plus ranibizumab: placebo and levosulpiride) will investigate the benefits of levosulpiride treatment on top of standard antiangiogenic therapy with ranibizumab in advanced DME. Although anti-VEGF agents are the firstline therapy for DME involving the central macula, approximately 50% of the patients do not respond suggesting the contribution of other vasoactive and proinflammatory mediators (28). Vasoinhibins block the vascular effects of VEGF and of other cytokines present in the vitreous of DR patients (17, 18, 20, 29), implying that levosulpiride may extend and potentiate the benefits of the anti-VEGF therapy. The comparison of serum and vitreous PRL levels between the groups 5 and 6 undergoing vitrectomy (DR: levosulpiride and placebo) will serve as a proof of principle that PRL enters the eye to counteract disease progression. Blood samples are drawn immediately prior to surgery and before the induction of anesthesia.

### Eligibility Criteria

#### Inclusion Criteria

- Age equal or greater than 40 years but no older than 69 years
- Male and female subjects with DME
- Male and female subjects with NPDR
- Male and female subjects with PDR undergoing medically prescribed vitrectomy
- Male and female subjects with DME, with or without DR, requiring ranibizumab
- Signed informed consent

#### Exclusion Criteria

- Not meeting inclusion criteria.
- History of ocular complications: severe myopia (>6 diopters), opacity of ocular media, glaucoma, ocular surgeries, and retinal detachment (except for PDR with vitrectomy)
- With retinal laser photocoagulation and/or intravitreal antiangiogenic therapy (<6 months before enrollment) (groups 1–4)
- With retinal laser photocoagulation and/or intravitreal antiangiogenic therapy (<6 weeks before enrollment) (groups 7 and 8)
- Prolactin serum levels >20 ng/mL
- Loss of kidney function (glomerular filtration rate <60 mL/min) for groups with DME, NPDR, and DME plus ranibizumab
- Loss of kidney function (glomerular filtration rate <30 mL/min) for groups with PDR undergoing vitrectomy
- Contraindications for the use of levosulpiride (Parkinson’s disease, epilepsy, breast cancer, alcoholism, hypokalemia)
- Conditions inducing hyperprolactinemia including pathologies (hypothyrodism, hepatic dysfunction, prolactinomas) and medication (antipsychotics, antidepressants, prokinetics, estrogens, other)

#### Elimination Criteria

- Worsening of disease requiring conventional treatment
- Not complying with study medication (monitored by drug tablet return and the measurement of the circulating levels of PRL)
- Hesitation to continue with study medication
- Voluntary withdrawal of consent
- Inability to continue in-hospital appointments
- Relocation to another state or country
- Missing outcome data

### Enrollment and Randomization

After potential participants have signed the informed consent, their eligibility is evaluated based on the inclusion/exclusion criteria (Figure 1). If eligible, their demographic characteristics, medical history, and physical examination are assessed; both eyes are evaluated for visual acuity [Early Treatment Diabetic Retinopathy Study (ETDRS) chart test], retinal thickness (non-invasive optical coherence tomography, OCT), and retinal microvascular abnormalities (fundus biomicroscopy and/or fluoroangiography). Laboratory tests are also carried out to determine circulating levels of PRL, glycated hemoglobin, alanine aminotransferase, thyroid-stimulating hormone, and creatinine (Figure 1). The evaluations confirm eligibility, allow for the distribution of patients, and set the baseline reference for treatment comparisons. DME is diagnosed by means of ophthalmoscopy and OCT measurements of central retinal thickness (30). Patients are classified as having NPDR and PDR according to the international clinical DR severity scale (31). DME and NPDR patients are randomized in a 1:1 ratio to receive levosulpiride (25-mg orally TID for 4 weeks) or placebo (lactose pill orally TID for 4 weeks). PDR patients requiring vitrectomy (due to non-clearing vitreous hemorrhage, tractional retinal detachment, and/or severe neovascular proliferation) are randomly split 1:1 to receive levosulpiride (25-mg orally TID) or placebo (lactose pill orally TID) 1 week before undergoing vitrectomy. Patients requiring antiangiogenic therapy for the treatment of clinically significant DME are randomized to receive levosulpiride (25-mg orally TID) or placebo (lactose pill orally TID) during the 12 weeks of standard ranibizumab intravitreal therapy (0.5 mg every 4 weeks) (Figure 1). In all groups, the study medication is taken on top of standard therapy for diabetes, blood pressure control, or other medical conditions. Specifically, this therapy includes metformin, sulfonylureas, dipeptidyl peptidase-4 inhibitors, acarbose, insulin, ACE inhibitors or angiotensin II receptor blockers, beta blockers, calcium antagonists, and/or diuretics, etc. In the case of adverse and intolerable effects of the study medication, the treatment is stopped and reported as a failure (32).

### Blinding

Physicians (primary investigators and investigators) enroll the patients. An investigator (coordinator) fully knowledgeable of all protocols, policies, procedures, and definitions in the study allocates the treatment. Patients, care providers, and evaluators are blind to treatment allocation. Unblinding is permissible only in the case of significant improvement or significant deterioration of primary outcome measures. Interim analyses will be performed by the study coordinator and the principal investigator not involved in the direct evaluation of patients.

### Outcome Measures

After enrollment, four study visits take place every 2 weeks during a follow-up period of 8 weeks in groups 1 and 2 (DME: placebo and levosulpiride) and groups 3 and 4 (NPDR: placebo and levosulpiride). For groups 7 and 8 (DME plus ranibizumab: placebo and levosulpiride) enrollment is followed by three visits, one every 4 weeks (at the time of ranibizumab intravitreal delivery). At each visit, the medical history, physical examination, adverse events, and primary and secondary outcomes are documented. For groups 5 and 6 (PDR requiring vitrectomy: placebo and levosulpiride), there is only one visit 1 week after enrollment, i.e., at the time of vitrectomy.

#### Objectives

The objectives of the study are to evaluate the effect of prolonged exposure to PRL induced by levosulpiride on DME and DR progression, evaluate the effect of levosulpiride as a combination therapy with ranibizumab on DME progression, and whether the actions of levosulpiride occur *via* the intraocular incorporation of PRL and its conversion to vasoinhibins. Therefore, the primary endpoints of the study medication are changes from baseline of ophthalmologic parameters and of PRL and vasoinhibin levels in the vitreous.

#### Primary Outcome Measures

1. Best-corrected visual acuity. Number of letters recognized in the ETDRS chart test after correcting for any refractive error (myopia, hyperopia, or astigmatism)
2. Retinal thickness evaluated by OCT imaging *via* qualitative and quantitative analyses. For qualitative analyses, OCT images approaching the histological level of retinal morphology are interpreted based on normal and diseased features (hyper-reflective or hypo-reflective lesions, shadowing, and anatomical changes). Quantitative analysis evaluates retinal reflective signals and their correlation with retinal morphology by computer image-processing algorithms (retinal thickness map, volume, area, 1-, 3-, and 6-mm ETDRS circle diameters)
3. Number, size, and location of retinal hard exudates and hemorrhages evaluated by fundus biomicroscopy
4. Location, area, and source of retinal microaneurisms, leakage area, cotton-wool spots, venous beading, micro-vascular, and vascular abnormalities evaluated by fundus fluorescein angiography imaging
5. Serum and vitreous levels of PRL (IMMULITE 2000XPI immunoassay system)
6. Vasoinhibin vitreous levels (immunoprecipitation-Western blot-densitometry)

#### Secondary Outcome Measures

The secondary endpoints evaluate the safety of the study medication by measuring:

1. Blood pressure
2. Blood glycated hemoglobin
3. Serum glucose
4. Serum creatinine
5. Hyperprolactinemia-related side effects documented at each participant visit (32)
6. Dopamine D2 receptor blocker side effects documented at each participant visit (32)

### Sample Size

The proposed sample size (Figure 1) was calculated according to expected differences in visual acuity, the main parameter of visual function. Using the formula for mean differences and the reported changes in visual acuity (33, 34), we considered a difference of 10 letters in the ETDRS chart test between baseline and after intervention, a SD of 10, a power 1 − β = 80%, and a type-I error rate of 5%. For PRL and vasoinhibin level outcome, sample size is in accord to experimental models of DR and clinical experience (14, 15). As the study progresses, the study may need to accommodate a different sample size to obtain clinically relevant and statistically significant differences, access to eligible patients, elimination of patients, etc.

### Data Collection and Analysis

The same physicians (ophthalmologists and endocrinologists) and laboratory technicians will collect and evaluate the data from all patients. Data are recorded in questionnaires, images and electronic quantitations (OCT, fundus biomicroscopy and fluoroangiography), and laboratory biochemical determinations. All images collected during the study are sent for re-assessment to a central evaluation center at the recruiting Institution to insure accurate evaluation. The coordinator of the study will continually examine the registry to insure all collected data are complete, accurate, and valid. Data logically inconsistent will be confronted with information in external database. Data collected on formatted paper forms are entered into a computer and electronic registries carefully reviewed by a third party to identify missing data, invalid or erroneous entries, and inconsistent data. Any data review activity and remediation efforts will be documented. Statistical methods evaluate continuous and categorical variables, the association between hormonal levels and outcome, and the relative contribution of confounding factors. The study data will be stored in the recruiting Institute through private accessing codes and kept for up to 10 years following the publication of the study. The data information will then be destroyed.

### Safety Considerations

Levosulpiride is clinically approved and available in different countries (Italy, Spain, Germany, Belgium, Mexico, South Korea, etc). It is used at low doses (25–75 mg/day) as a prokinetic agent for the treatment of dysmotility-like functional dyspepsia (35, 36). It penetrates the blood–brain barrier poorly (37) so higher doses (800–2400 mg/day) are required for its use as an anti-depressant and anti-psychotic agent (38). Duration of treatment ranges from 1 week to more than 2 years (23). Low doses of levosulpiride are well tolerated during both acute and chronic administration. Heart rate and electrocardiogram are not altered (35) and sedation is rare (36). As the pituitary is outside the blood–brain barrier, low doses of levosulpiride increase serum PRL (39). Adverse effects of hyperprolactinemia include decreased libido, amenorrhea and infertility, breast engorgement and lactation, and gynecomastia (40). In a multicenter, double-blind, placebo-controlled trial of 408 dyspeptic patients receiving oral levosulpiride (25-mg TID for 4 weeks) the total incidence of hyperprolactinemia related disorders was 7% in female patients (41). More recently, a study in 342 dyspeptic female and male patients, using the same levosulpiride treatment, reported 40 patients (11%) with adverse effects (26.7% galactorrhea, 17.8% somnolence, 11% fatigue, and 11.5% headache), none of whom abandoned the study due to side effects (36).

### Current Status

Patients started enrolling in this trial on May 2016. As of January 2018, 270 patients were screened and 63 identified as candidates signed the informed consent. Later nine patients were excluded (1 due to hyperprolactinemia, 6 because of glomerular filtration rates <30 mL/min, 1 due to glaucoma, and 1 requiring laser therapy). From the remaining 54 study subjects, 9 dropped-out after randomization (3 did not comply with the study medication, 2 missed outcome data, 1 required conventional therapy, and 3 vitrectomies were rescheduled as patients forgot to take hypertension medication before surgery and had very high blood pressure values), and 45 completed the study. Relevant protocol modifications will be registered at ClinicalTrials.gov. The protocol last amendment was registered on October 3, 2017 and consisted in the inclusion of DME patients receiving placebo or levosulpiride on top of standard antiangiogenic therapy with ranibizumab.

## Discussion

### Clinical Implications

Diabetes mellitus affects nearly 10% of world population and all diabetic individuals are at risk of developing DR and DME. Current standard treatments (laser photocoagulation, vitrectomy, and anti-VEGF therapies) reduce the risk of blindness and vision loss in DR and DME but seldom restore normal vision, are destructive, or their intravitreal delivery can lead to ocular complications over time (7). Moreover, the feasibility of providing these therapies in a timely, effective, and cost-accessible manner is challenging, particularly in developing countries. Our study examines for the first time the efficacy and safety of an oral treatment for DR and DME that uses levosulpiride, a medication prescribed to treat diabetic gastroparesis. The rationale underlying this treatment is based on the fact that levosulpiride effectively induces hyperprolactinemia, and preclinical studies show that hyperprolactinemia leads to the accumulation of intraocular vasoinhibins able to counteract diabetes-induced retinal vascular alterations by blocking not only the effects of VEGF (17, 18), but of other contributing factors in the vitreous of RD and DME patients (20, 29). Historically, there are several reports in which a rise in systemic PRL levels, and a subsequent rise in retinal vasoinhibins, may have contributed to the improvement of retinal vascular alterations in patients with diabetes. A context between pituitary gland function and DR was initially reported in 1953, when a recovery from retinopathy was observed after the patient developed Sheehan’s syndrome (postpartum pituitary gland necrosis) (42). In this case, and later in cases in which pituitary stalk sections were performed with a therapeutic intention (today considered unethical), the improvement of retinopathy was interpreted to be a consequence of declining GH and IGF-I levels. However, there was no consistent association between GH/IGF-I levels and DR in subsequent investigations (43). Hence, the beneficial effect on retinopathy associated with pituitary stalk section or other alterations of pituitary function may have been, in part, also explained by a rise of retinal vasoinhibins as a consequence of an increased PRL secretion. This may also apply for a case in which a stabilization of retinopathy was reported in association with the onset of hyperprolactinemia due to a prolactinoma (44) (For a more detailed discussion, the reader is referred to the review cited in reference (13)).

Moreover, diabetes damages all retinal cell types (4, 45) and PRL, itself, ameliorates neuronal, glial, and pigment epithelium dysfunction in the retina (22, 46). Therefore, levosulpiride is a less-invasive treatment with potential for controlling diabetic eye disease and for improving the efficacy of anti-VEGF medication, particularly in those patients with a limited response.

### Strengths and Limitations of the Study

A strength of the study is the use of an oral therapy with a medication regimen that has been proven safe in diabetic patients (36). Adverse side effects (galactorrhea, somnolence, fatigue, and headache) are mild and rare, decline with time, and usually do not result in patients abandoning the study (36). In our protocol, side effects and risks are further minimized by frequent medical supervision (every 2 weeks) and short treatment duration (2 months) when administered as monotherapy. Its use as a combination therapy with ranibizumab, not only reduces ophthalmological risks, as patients are being treated with the standard therapy, but extends possible beneficial effects to patients with clinically significant DME, the major diabetic population requesting eye-care in underdeveloped countries. The absence of levosulpiride clinical studies in diabetic patients with retinal and renal microvascular complications limits the study design. We are aware that the follow-up period may be too short to provide specific treatment recommendations, but the short-term results could encourage further prospective studies with different treatment regimen and longer follow-up. Levosulpiride retains its potency to induce hyperprolactinemia at very low doses (38), so smaller doses coupled to long-term treatments can be a desirable therapy.

## Conclusion

This study investigates the therapeutic potential of levosulpiride by itself and as an add-on treatment in addition to standard anti-VEGF therapy to improve DR and DME. Being an oral-treatment, it is less invasive than current therapies, and it has an adequate level of safety supported by its recommended use for diabetic gastroparesis. No clinical work supports its clinical use against diabetic eye disease, but preclinical evidences are ample and strong. If shown to be effective, it will have the potential to impact on the loss vision in persons with diabetes.

## Dissemination

After completing the study, all data, including beneficial and adverse events, of trial intervention will be communicated at scientific meetings and published in indexed peer-reviewed journals. If shown to be effective, the therapy program will be made available to the general public in Mexico in an appropriate manner.

## Ethics Statement

The Ethics Committees of the National University of Mexico (Universidad Nacional Autónoma de México, UNAM) and the Instituto Mexicano de Oftalmología, I.A.P. approved the study. All procedures are performed in accord with the Declaration of Helsinki and the study has been registered on ClinicalTrials.gov (NCT03161652). Written informed consent to participate is obtained for each subject prior to the study.

## Author Contributions

MLR-O, principal investigator, endocrinologist, supervisor, conceiving, designing, and carrying out the study, critically revising the manuscript. RG-F, principal investigator, ophthalmologist (retina and vitreous specialist), supervisor, designing, and carrying out the study, critically revising the manuscript. CDN-A, investigator, coordinator ensuring compliance, planning, and carrying out the study. XM-L, investigator, ophthalmologist (retina and vitreous specialist) planning, and carrying out the study. PR-N, investigator, ophthalmologist (retina and vitreous specialist) planning, and carrying out the study. WH, investigator, coordinator ensuring compliance, planning, and carrying the study. EL-S, supervisor, designing and revising the study. TB, supervisor, revising the work. GM de la E, supervisor, designing, and critically revising the study. JT, principal investigator, supervisor, designing the study, and critically revising the manuscript. CC, sponsor and principal investigator, coordinator and supervisor, conceived and designed the study, and wrote the manuscript. All authors agree to be accountable for the content of the work and approved the submitted version of the manuscript.

## Conflict of Interest Statement

All authors declare no conflict of interest or financial interest, arrangement, or affiliation with the manufacturer of the study drug.

## Abbreviations

DME, diabetic macular edema; DR, diabetic retinopathy; ETDRS, Early Treatment Diabetic Retinopathy Study; NPDR, non-proliferative diabetic retinopathy; OCT, optical coherence tomography; PDR, proliferative diabetic retinopathy; PRL, prolactin; TID, three times a day; VEGF, vascular endothelial growth factor.
